# Platelet activation independent of pulmonary inflammation contributes to diesel exhaust particulate-induced promotion of arterial thrombosis

**DOI:** 10.1186/s12989-016-0116-x

**Published:** 2016-02-09

**Authors:** Caroline M. Tabor, Catherine A. Shaw, Sarah Robertson, Mark R. Miller, Rodger Duffin, Ken Donaldson, David E. Newby, Patrick W. F. Hadoke

**Affiliations:** 1 Univeristy/ BHF Centre for Cardiovascular Sciences, Edinburgh, EH16 4TJ UK; 2 Centre for Inflammation Research, The Queen’s Medical Research Institute, Universiyt of Edinburgh, Edinburgh, EH16 4TJ UK

**Keywords:** Air pollution, Diesel exhaust particles, Thrombosis, Platelet activation, Fibrinolysis, Inflammation

## Abstract

**Background:**

Accelerated thrombus formation induced by exposure to combustion-derived air pollution has been linked to alterations in endogenous fibrinolysis and platelet activation in response to pulmonary and systemic inflammation. We hypothesised that mechanisms independent of inflammation contribute to accelerated thrombus formation following exposure to diesel exhaust particles (DEP).

**Methods:**

Thrombosis in rats was assessed 2, 6 and 24 h after administration of DEP, carbon black (CB; control carbon nanoparticle), DQ12 quartz microparticles (to induce pulmonary inflammation) or saline (vehicle) by either intra-tracheal instillation (0.5 mg, except Quartz; 0.125 mg) or intravenous injection (0.5 mg/kg). Thrombogenicity was assessed by carotid artery occlusion, fibrinolytic variables and platelet-monocyte aggregates. Measures of inflammation were determined in plasma and bronchoalveolar lavage fluid. Tissue plasminogen activator (t-PA) and plasminogen activator inhibitor (PAI)-1 were measured following direct in vitro exposure of human umbilical vein endothelial cells (HUVECs) to DEP (10–150 μg/mL).

**Results:**

Instillation of DEP reduced the time to thrombotic occlusion in vivo, coinciding with the peak of DEP-induced pulmonary inflammation (6 h). CB and DQ12 produced greater inflammation than DEP but did not alter time to thrombotic occlusion. Intravenous DEP produced an earlier (2 h) acceleration of thrombosis (as did CB) without pulmonary or systemic inflammation. DEP inhibited t-PA and PAI-1 release from HUVECs, and reduced the t-PA/PAI-1 ratio in vivo; similar effects in vivo were seen with CB and DQ12. DEP, but not CB or DQ12, increased platelet-monocyte aggregates.

**Conclusion:**

DEP accelerates arterial thrombus formation through increased platelet activation. This effect is dissociated from pulmonary and systemic inflammation and from impaired fibrinolytic function.

**Electronic supplementary material:**

The online version of this article (doi:10.1186/s12989-016-0116-x) contains supplementary material, which is available to authorized users.

## Background

The association between air pollution and cardiovascular disease is well-established [1], with exposure to traffic-derived pollutants implicated in acute myocardial infarction [2, 3]. The adverse cardiovascular effects of airborne pollution have been attributed to combustion-derived particulate matter (PM), especially ultrafine particles from diesel exhaust (DEP), that has a large reactive surface area and can penetrate deep within the lung (reviewed in [4]). Recent investigations suggest that DEP [5] and ambient particulate matter [6, 7] increase cardiovascular events by accelerating thrombosis [2].

The mechanisms responsible for PM-induced enhancement of thrombosis remain controversial. Diesel exhaust inhalation in humans produced endothelial cell dysfunction 2–24 hours after exposure [8–10], accompanied by impaired endogenous fibrinolysis [8], increased thrombogenicity and platelet activation [5]. These effects could be directly attributed to the exhaust particles as they were markedly reduced following removal of particles from the exhaust [11, 12]. Investigations using appropriate animal models support the contention that DEP increases the likelihood of thrombus formation [13, 14]. However, whilst several different mechanisms for enhanced coagulation in response to particles have been implicated (including impaired fibrinolysis [7] and platelet activation [7, 15, 16]), there remains much disagreement. A recent study in mice, indicated that increased thrombus formation could be attributed to PM-induced pulmonary and systemic inflammation via multiple mechanisms [7]; including an increased tumour necrosis factor (TNF)-α-dependent impairment of fibrinolysis and an interleukin (IL)-6-dependent activation of coagulation. However, whether the cardiovascular effects of DEP require direct interaction with the lung is uncertain since intravenous (i.v.) injection caused systemic and pulmonary inflammation associated with alterations in the cardiovascular system in spontaneously hypertensive rats [17].

There is also some evidence that DEP can, at least acutely, increase coagulation independent of pulmonary inflammation. Inhibition of pulmonary inflammation 1 h after exposure of hamsters to DEP had no effect on coagulation or platelet activation, whereas the same intervention did reduce DEP-mediated enhancement of coagulation 3 and 24 h after exposure [18]. It was suggested that penetration of DEP, or components of DEP, into the blood could account for this early pro-thrombotic effect. This would certainly be consistent with the demonstration that direct exposure of blood to DEP can enhance thrombus formation ex vivo [1].

Whilst previous investigations have considered the role of pulmonary inflammation, uncertainty remains about its influence on the pro-thrombotic actions of DEP. The current investigation addressed the hypothesis that exposure to DEP induces an increase in thrombosis independent of pulmonary inflammation. This was addressed in vivo by using (1) intra-pulmonary and (2) intravenous administration of DEP or appropriate ‘control particles’ (carbon black, DQ12 quartz). These studies were complemented by in vitro experiments to determine whether direct exposure to DEP alters endogenous fibrinolysis in endothelial cells.

## Results

### IT instillation of particles

IT instillation (500 μg DEP/rat) resulted in successful administration of DEP, with particle aggregates evident in alveoli (but not in liver, kidney or carotid artery thrombi) collected 2 or 6 h after administration (data not shown).

#### Pulmonary & systemic inflammation

**Fig. 1:**
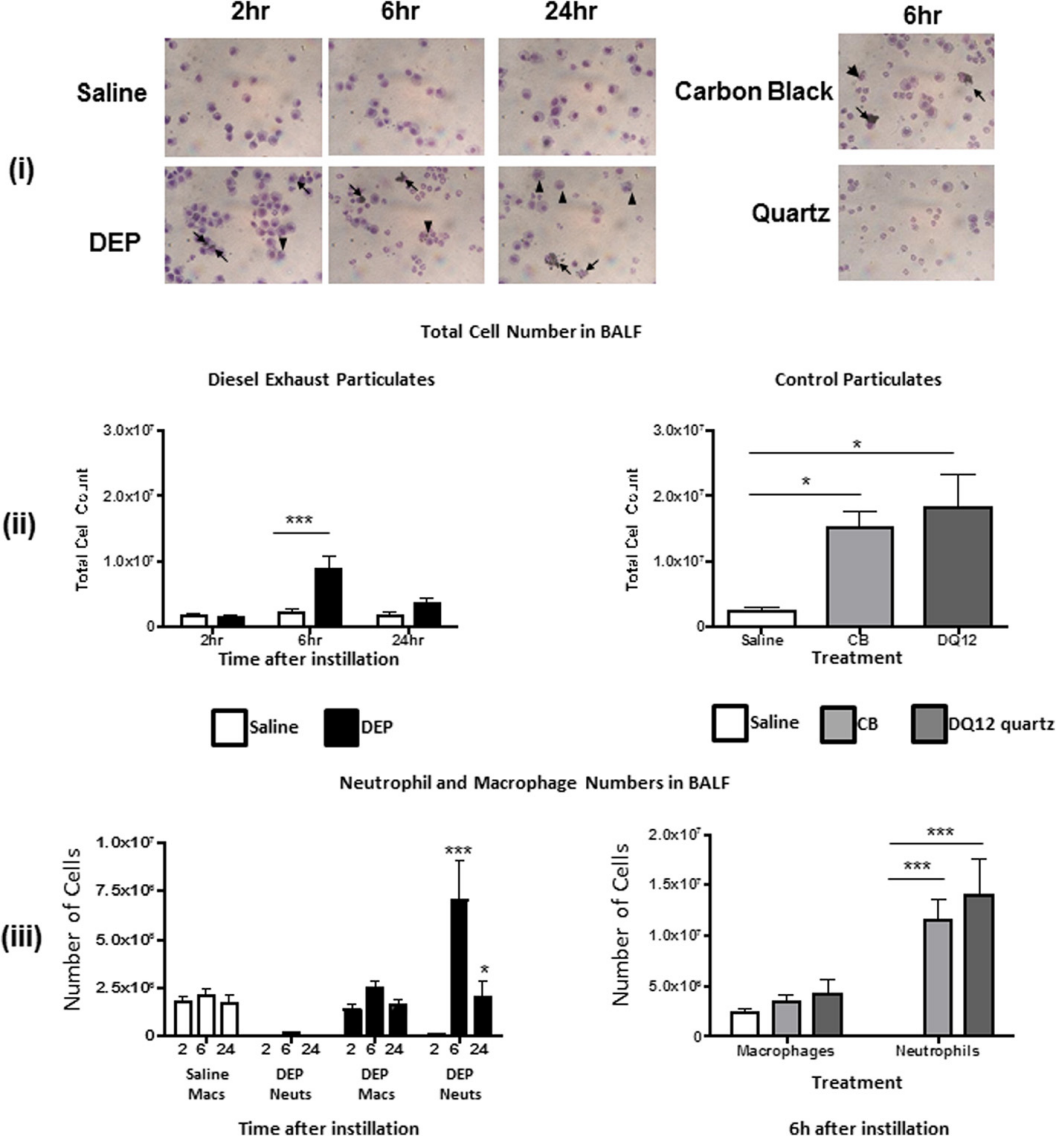
Particle instillation causes pulmonary neutrophil accumulation. (**i**) Monocytes and neutrophils in bronchoalveolar lavage fluid (BALF) were identified by nuclear morphology in haematoxylin and eosin-stained cytospins after in vivo administration of diesel exhaust (DEP; 2, 6 or 24 h) or control (*carbon black* (CB); DQ12 quartz; 6 h) particle solutions. DEP and CB were associated with inflammatory cells (*arrows*) or phagocytosed by macrophages (*arrow heads*). There was no evidence of phagocytosis of quartz particles. Magnification ×400. (**ii**) Instillation of DEP (0.5 mg/rat; *black columns*) caused a transient increase in total cell number that peaked 6 h following administration. CB (0.5 mg/rat; *light grey columns*) or DQ12 quartz (0.125 mg/rat; *dark grey columns*) also increased cell number 6 h after instillation. (**iii**) Increases in total inflammatory cell number were due to an increase in neutrophils. Data are mean ± s.e.mean (*n* = 6) and were compared with appropriate control using Student’s unpaired *t*-test. *P < 0.05, ***P < 0.001

**Fig. 2:**
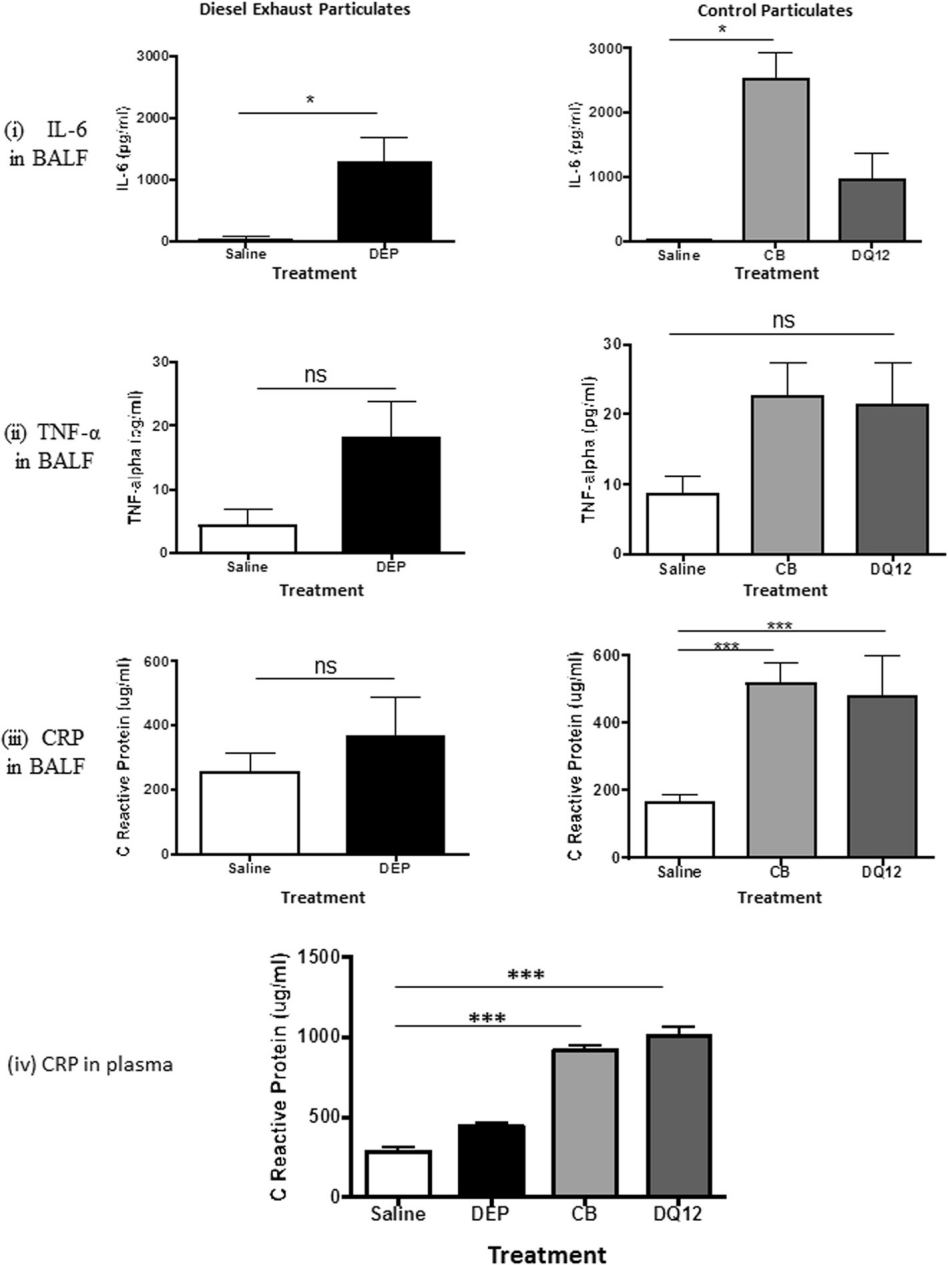
Influence of particle instillation on pro-inflammatory cytokines. Instillation of diesel exhaust particles (DEP) increased IL-6 concentrations in bronchoalveolar lavage fluid (BALF) (**i**), but apparent increases of tumour necrosis factor (TNF)-α (**ii**) and C-reactive protein (CRP) (**iii**) did not achieve significance. Carbon black (CB) increased concentrations of IL-6 and CRP, whilst DQ12 quartz increased CRP. (**iv**) In plasma, carbon black (CB; *light grey columns*) or DQ12 quartz (*dark grey columns*), increased concentrations of CRP compared with controls (*white columns*), whereas DEP (*black columns*) did not. Data are mean ± s.e.mean (*n* = 6) and were compared with relevant control using Student’s unpaired *t*-test or one-way ANOVA, as appropriate. *P < 0.05, ***P < 0.001. ns = not significant

IT instillation of DEP, CB or DQ12 quartz induced clear, transient pulmonary inflammation with a marked influx of cells (predominantly neutrophils), peaking 6 h post instillation (Fig. 1). The responses produced by CB and DQ-12 particles were greater than those produced by DEP (Fig. 1). Cellular infiltration was accompanied by increased concentrations of interleukin (IL)-6 in bronchoalveolar lavage fluid (BALF) 6 h after instillation (Fig. 2i), although apparent increases in tumour necrosis factor (TNF)-α (Fig. 2ii) and C-reactive protein (CRP; Fig. 2iii) did not achieve statistical significance. The indication that the pulmonary inflammation produced by control particles was more marked than that induced by DEP was supported by the demonstration that CB increased both IL-6 (Fig. 2i) and CRP (Fig. 2iii), whilst DQ12 quartz increased CRP concentrations in BALF (Fig. 2iii). Furthermore, control particles induced systemic inflammation: concentrations of CRP in plasma (6 h) were increased by IT instillation of CB or DQ12 quartz, but unaltered after IT instillation of DEP (Fig. 2v). Plasma concentrations of IL-6 and TNFα were not affected by IT instillation of any of the particles (data not shown).

#### Thrombus formation

**Fig. 3:**
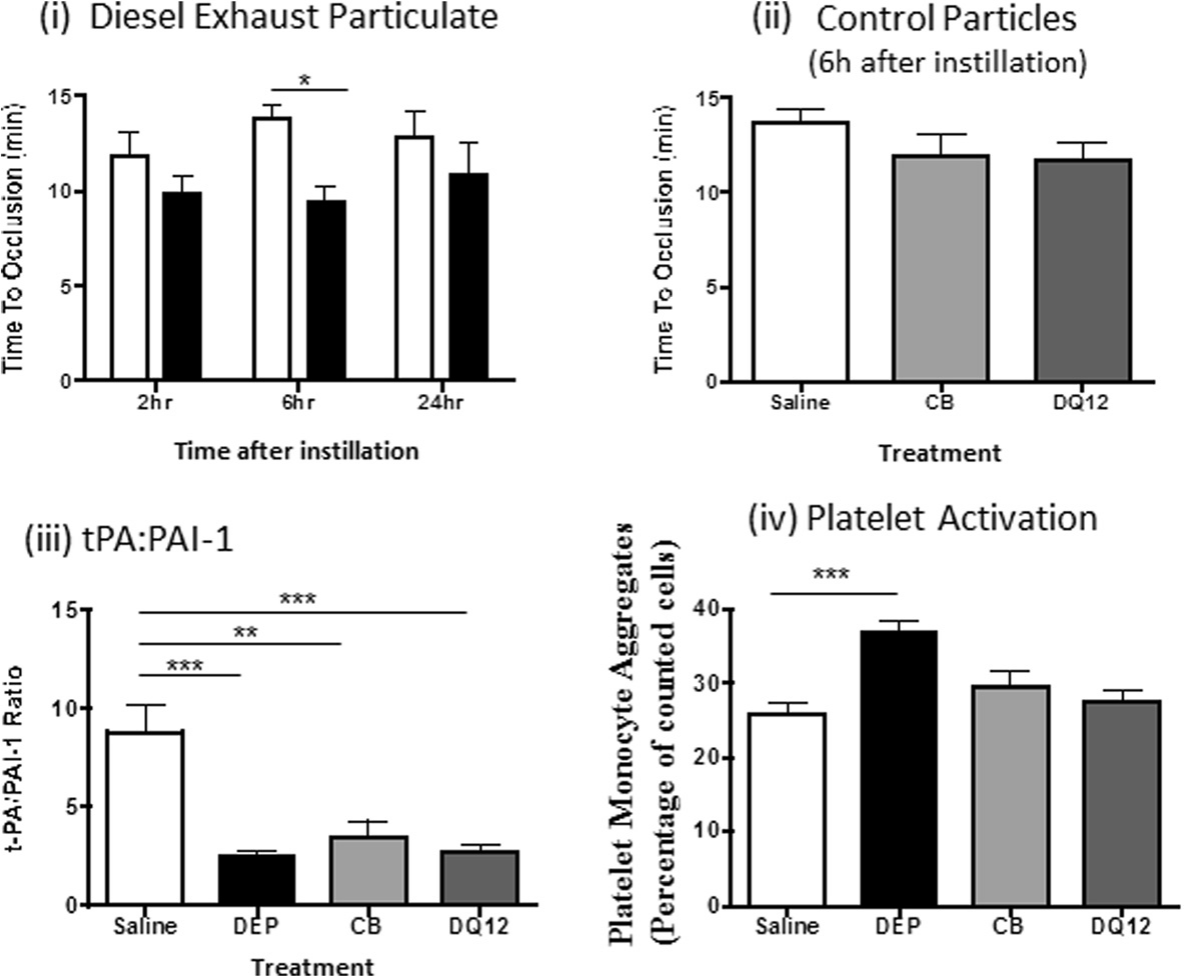
Influence of particle instillation on thrombogenesis. Diesel exhaust (DEP; *black colums*) or control (*carbon black* (CB), *light grey columns*; DQ12 quartz, *dark grey columns*) particles were administered by intra-tracheal instillation 2, 6 or 24 h before induction of arterial thrombosis. (**i**) Instillation of DEP (0.5 mg/rat) reduced time to occlusion (*P* < 0.05), compared with the vehicle (*open columns*) 6 h after administration. Apparent reductions in time to occlusion 2 h and 24 h after administration did not achieve significance. (**ii**) Control particles (CB (0.5 mg/rat) or DQ12 quartz (125 mg/rat;)) did not alter time to occlusion 6 h after instillation. (**iii**) The ratio of tissue plasminogen activator (t-PA) to plasminogen activator inhibitor (PAI)-1 was reduced in the plasma of rats following intra-tracheal (IT) instillation of DEP, CB or DQ12 quartz. (**iv**) IT instillation of DEP, but not CB or DQ12 quartz increased platelet-monocyte interactions. Data are mean ± s.e.mean (*n* = 6) and were compared with relevant controls using one-way ((**ii**, (**iii**), (**iv**)) or two way (**i**) ANOVA. *P < 0.05, **P < 0.01 and ***P < 0.001

FeCl_3_ produced thrombus formation, leading to total occlusion (cessation of blood flow) of the carotid artery after 12–15 min in vehicle-treated rats. IT instillation of DEP (Fig. 3i) produced a pattern of reduced time-to-occlusion at all three time-points assessed (2, 6 & 24 h), with the difference achieving significance 6 h after exposure (DEP 9.4 ± 0.8 min vs vehicle 13.8 ± 0.7 min; *n* = 6). In contrast, neither CB nor DQ12 quartz accelerated thrombus formation (Fig. 3ii).

#### Endogenous fibrinolysis

Plasma PAI-1 and t-PA concentrations were measured to determine whether altered endogenous fibrinolysis could account for the accelerated thrombosis induced by DEP. These measurements demonstrated that IT instillation of DEP reduced plasma t-PA and increased PAI-1 concentrations, reducing the t-PA:PAI-1 ratio (Fig. 3(iii)). Instillation of CB or DQ12 quartz produced a similar impairment in fibrinolytic function (Fig. 3(iii)).

#### Platelet-monocyte aggregation

Platelet-monocyte aggregation was increased 6 h after IT instillation of DEP (Fig. 3(iv)). In contrast, instillation of CB or DQ12 Quartz had no effect.

### IV injection of particulate

Successful administration of particles to the circulation was confirmed by the identification of DEP aggregates in sections of liver (although not in kidney) 2 h after intravenous injection (data not shown). No particles were identified in these organs 6 or 24 h after injection.

#### Absence of pulmonary and systemic inflammation

**Fig. 4:**
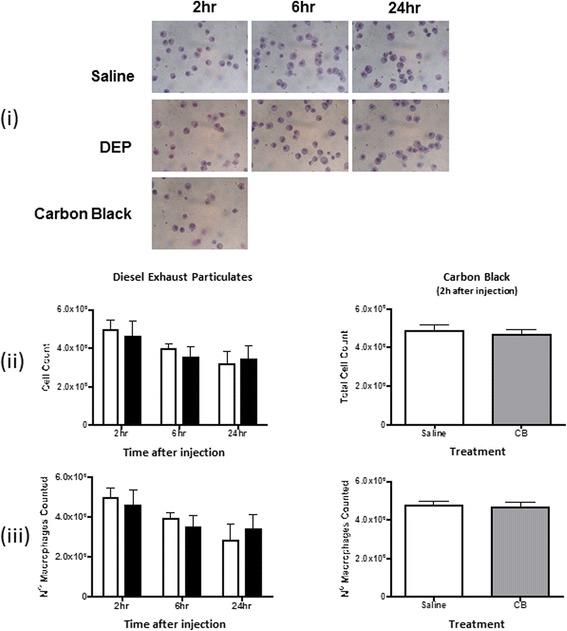
Intravenous injection of diesel exhaust particles or carbon black does not cause pulmonary neutrophil accumulation. (**i**) Example cytospins of cells within bronchoalveolar lavage fluid (BALF) recovered 2, 6 or 24 h following injection of diesel exhaust particles (DEP) or 2 h after intravenous injection of carbon black (CB) solutions. Staining: haematoxylin and eosin, magnification ×400. (**ii**) Systemic injection of DEP (*black bars*) or CB (*light grey bars*) did not increase the total number of cells in BALF. (**iii**) Differential analysis confirmed that the majority of cells were macrophages, both in saline-treated (*white columns*) and in particle-treated rats. Data are mean ± s.e.mean (*n* = 6). All comparisons with saline-treated control were statistically non-significant; *P* > 0.05 using two-way ANOVA (DEP vs Saline) or Student’s unpaired *t*-test CB vs Saline)

In contrast to IT instillation, i.v. injection of DEP or CB did not cause pulmonary inflammation. There was no influx of inflammatory cells into the lung (Fig. 4i), and no change in the inflammatory cell populations (Fig. 4ii & iii) or the cytokine concentrations in BALF: TNFα (Additional file 1: Figure S1i), CRP (Additional file 1: Figure S1ii). IL-6 concentrations were below the limit of detection (data not shown). In addition, there was no evidence of systemic inflammation as plasma concentrations of CRP, IL-6 and TNFα were unaffected by i.v. DEP. Interestingly, plasma CRP (but not IL-6 or TNFα; data not shown) was increased 2 h after injection of CB (Additional file 1: Figure S1iii).

#### Thrombus formation

**Fig. 5:**
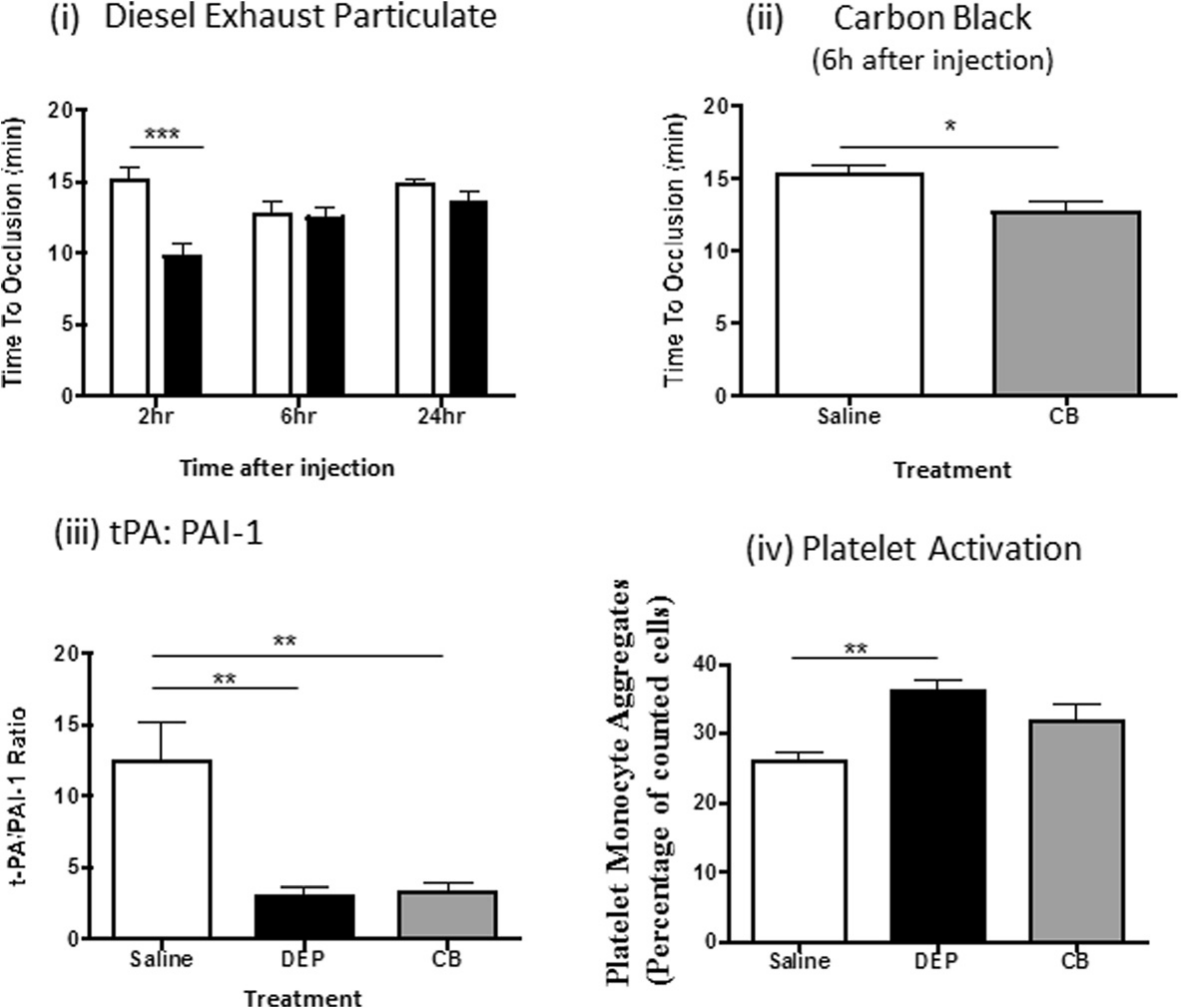
Influence of particle injection on thrombogenesis. Diesel exhaust (DEP; *black columns*) or control particles (*carbon black* (CB, *grey columns*)) were administered by intravenous injection 2, 6 or 24 h before induction of arterial thrombosis. (**i**) Injection of DEP (0.5 mg/rat) reduced time to occlusion (*P* < 0.05), compared with saline-treated controls (*open columns*) 2 h (but not 6 or 24 h) after administration. (**ii**) Injection of carbon black (CB (0.5 mg/rat)) produced a similar reduction in time to occlusion (*P* < 0.05). (**iii**) The ratio of tissue plasminogen activator (t-PA) to plasminogen activator inhibitor (PAI)-1 was reduced in the plasma of rats after intravenous injection (IV; 2 h post exposure) of DEP or CB. (**iv**) Platelet-monocyte interaction was increased following IV administration of DEP but not CB. Data are mean ± s.e.mean (*n* = 6) and were compared with relevant controls using two way ANOVA (DEP vs Saline) or Student’s unpaired *t*-test (CB vs Saline). *P < 0.05, **P < 0.01, ***P < 0.001

In the FeCl_3_-induced thrombosis model, DEP (0.5 mg/kg, i.v.) produced a transient reduction in time-to-occlusion 2 h following administration, which was no longer evident after 6 or 24 h (Fig. 5ii). The control particles, CB, also produced a small reduction in time-to-occlusion at the 2 h time-point (Fig. 5ii). No particles were identified by histological analysis of the thrombosed vessel.

#### Endogenous fibrinolysis

Injection (i.v) of DEP or CB reduced plasma t-PA and increased PAI-1 concentrations, reducing the t-PA:PAI-1 ratio (Fig. 5(iii)).

#### Platelet-monocyte aggregation

Platelet-monocyte aggregation was increased 2 h after i.v. injection, whereas exposure to CB had no effect (Fig. 5(iv)).

### In vitro exposure of HUVECs to DEP

In order to determine whether DEP can directly alter the fibrinolytic system in endothelial cells, cultured human umbilical vein endothelial cells (HUVECs) were exposed (16 h) to DEP (10–150 μg/mL) or vehicle, with or without thrombin (1.0 U/ml, 24 h) stimulation.

**Fig. 6:**
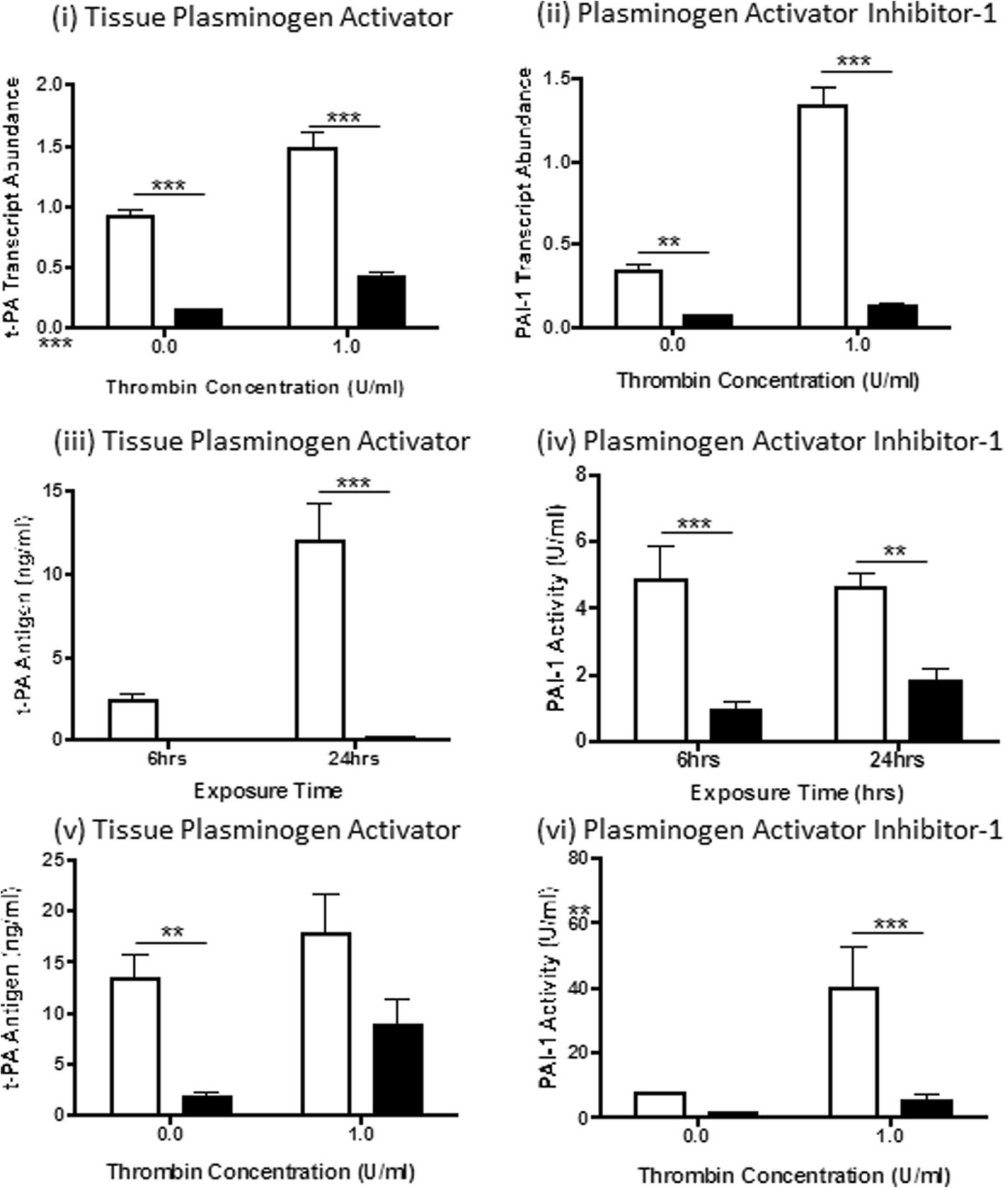
Diesel exhaust particles alter the endogenous fibrinolytic system in HUVECs. Human umbilical vein endothelial cells were exposed to diesel exhaust particles (DEP; 100 μg/ml, 16 h, *black columns*) or vehicle (*white columns*) then incubated in the presence or absence of thrombin (1.0 U/ml, 24 h). Pre-exposure to DEP reduced expression of (**i**) tissue plasminogen activator (t-PA) and (**ii**) plasminogen activator inhibitor (PAI-1). Exposure to DEP (150 μg/ml; 6–24 h) also reduced (**iii**) t-PA antigen concentrations and (**iv**) PAI-1 activity in culture media. Stimulation with thrombin did not significantly increase t-PA antigen levels in media (**v**) but increased PAI-1 activity (**vi**). Thrombin-stimulated PAI-1 activity was suppressed in cells pre-incubated with DEP. Data are mean ± s.e.mean (*n* = 6). Comparisons were made using Student’s unpaired *t*-test; **P < 0.01, ***P < 0.001

Exposure to DEP was not cytotoxic as treatment had no effect on cell viability as measured by LDH assay (Additional file 2: Figure S2). Pre-exposure to DEP reduced expression of t-PA (Fig. 6i) and PAI-1 (Fig. 6ii). Exposure to DEP (150 μg/ml; 6–24 h) also reduced t-PA antigen concentrations (Fig. 6iii) and PAI-1 activity (Fig. 6iv) in culture media. Stimulation with thrombin had no effect on t-PA antigen levels (Fig. 6v) but increased PAI-1 activity (Fig. 6vi) in media, an effect that was attenuated in the presence of DEP.

## Discussion

This investigation addressed the hypothesis that exposure to DEP induces accelerated thrombus formation independent of pulmonary inflammation. This was achieved by comparing the effects of pulmonary and systemic DEP administration with appropriate control particles (CB, a “clean” carbon particle to control for the nano-carbon core of DEP and DQ12 quartz, a large pro-inflammatory particle that will not translocate into the blood [19, 20]). Instillation and injection of DEP (and other nanoparticles) have been shown to increase pulmonary and systemic inflammation, with circulating levels of IL-6, TNFα and leukotriene-B4 elevated in the rat [14, 20–24]. The current investigation demonstrated that control particles caused pulmonary and/or systemic inflammation but did not promote thrombosis. In contrast, DEP-induced acceleration of thrombus formation was evident in the absence of pulmonary or systemic inflammation. The outcomes of these experiments are summarised in Table 1.

**Table 1:** Comparative summary of data from experiments using I.T. instillation (in vivo), I.V. injection (in vivo) and cultured endothelial cells (in vitro)

			Particulate		
		Time (hours)	DEP	CB	DQ12
I.T. instillation	Pulmonary cells	2	NSD	------	------
(In vivo)		6	↑	↑↑	↑↑
		24	NSD	------	------
	Pulmonary IL-6	6	↑	↑	NSD
	Pulmonary TNFα	6	NSD	NSD	NSD
	Pulmonary CRP	6	NSD	↑↑	↑↑
	Plasma IL-6	6	NSD	NSD	NSD
	Plasma TNFα	6	NSD	NSD	NSD
	Plasma CRP	6	NSD	↑↑	↑↑
	Thrombosis	2	NSD	------	------
		6	↑	NSD	NSD
		24	NSD	------	------
	tPA:PAI-1	6	↓↓	↓↓	↓↓
	PMA		↑↑	NSD	NSD
I.V. injection	Pulmonary cells	2	NSD	------	------
(In vivo)		6	NSD	NSD	------
		24	NSD	------	------
	Pulmonary IL-6	2	L.O.D	L.O.D.	------
	Pulmonary TNFα	2	NSD	NSD	------
	Pulmonary CRP	2	NSD	NSD	------
	Plasma IL-6	2	NSD	NSD	------
	Plasma TNFα	2	NSD	NSD	------
	Plasma CRP	2	NSD	↑↑	------
	Thrombosis	2	↑	------	------
		6	NSD	NSD	------
		24	NSD	------	------
	tPA:PAI-1	6	↓↓	↓↓	------
	PMA	24	↑	NSD	------
Cultured HUVECs	tPA transcripts	Basal	↓	------	------
In vitro		Stimulated	NSD	------	------
	PAI-1 transcripts	Basal	NSD	------	------
		Stimulated	↓	------	------
	tPA antigen	6	NSD	------	------
		24	↓↓	------	------
	PAI-1 activity	6	↓↓	------	------
		24	↓↓	------	------

*CB* carbon black, *CRP* C-reactive protein, *DEP* diesel exhaust particles, *DQ12* quartz particles, *HUVEC* human umbilical vein endothelial cells, *IL-6* interleukin 6, *PAI-1* plasminogen activator inhibitor, *PMA* platelet monocyte aggregation, *TNFα* tumour necrosis factor-α, *tPA* tissue plasminogen activator

NSD = no significant difference. ----- = not tested (note, DQ12 was not tested by iv injection or cultured endothelial cells as this particle is unlikely to cross from the lungs into the circulation due to its micrometre size)

### Inflammation and accelerated thrombosis following intra-tracheal instillation of DEP

Intra-tracheal (IT) instillation has been used extensively to investigate the effects of pulmonary exposure to a number of different particles [13, 24, 25]. It provides excellent alveolar penetration and distribution, and allows direct comparison of different particle types, with increased thrombus formation and platelet activation reported after pulmonary instillation of silica [26], titanium dioxide (TiO2) nanorods [27], ambient particulate matter [14], and carbon nanotubes [28]. Instillation has the advantage of ensuring direct administration of a controlled particle dose, whereas with inhalation there can be uncertainty about the proportion of particles that actually reaches the lungs (due to retention in the nasal cavity) [29]. IT instillation or inhalation of combustion-derived particles produce similar effects on thrombus formation in vivo [7]. The dose of DEP used for IT instillation (500 μg DEP/rat) is comparable with previous studies in animals (5–500 μg/animal for hamsters [13, 21, 24]) exploring particle activity, and can be used to consistently induce pulmonary inflammation.

The accelerated thrombosis induced 6 h after IT instillation of DEP was consistent with the pro-thrombotic effect described both in clinical [5, 8–10], and in animal (hamsters [13, 21, 30], mice [14, 22], or rats [23, 31]) studies. This enhanced thrombus formation was co-incident with the peak in pulmonary inflammation. Whilst this would be consistent with thrombosis increasing as a consequence of pulmonary inflammation [7], neither CB nor DQ12 quartz (which both induced inflammation) accelerated thrombus formation. This contrasts with the relationship between increased thrombosis and pulmonary inflammation reported for a number of different particles (silica [26], TiO2 [27], carbon nanotubes [28], DEP [13, 22]). More recent evidence, however, has suggested that CB-induced thrombogenic effects are independent of pulmonary and systemic inflammation [32].

The possibility that increased thrombosis is the result of impaired endogenous fibrinolysis has been indicated in a number of investigations [3]. Endothelial t-PA release was reduced in healthy volunteers [8] and in men with stable coronary heart disease [9] 2–6 hours after exposure to DE. The particulate constituents of these emissions contributed to the impaired t-PA release [11]. Impaired endogenous fibrinolysis has also been reported in some (but not all [33, 34]) studies in animals, with increased PAI-1 and reduced tissue factor pathway inhibitor following exposure to urban particulate matter [7, 14, 35]. The timing of these changes suggests involvement of an inducible pathway or changes in protein synthesis. Whether these changes are secondary to effects in the lung or are a consequence of direct interaction of DEP with the arterial wall has not been determined. The presence of pulmonary and systemic inflammation following particle administration may be expected to influence fibrinolysis since chronic inflammation is associated with impairment of the endogenous fibrinolytic system in vivo [36] and in cultured endothelial cells [37]. Furthermore, reduction of systemic inflammation (with the antioxidant ascorbic acid) restored fibrinolytic function in chronic smokers [38]. However, whereas our demonstration that IT instillation of DEP alters the endogenous fibrinolytic system is consistent with the impaired fibrinolysis reported after inhalation of DE in humans [8], the use of control particles indicated that altered fibrinolysis could not be the sole cause of increased thrombosis following instillation. CB or DQ12 quartz, which produced a similar impairment in fibrinolytic function, did not alter thrombus formation. These results suggest, therefore, that altered endogenous fibrinolysis may be just one component of DEP-mediated acceleration of thrombosis. Interestingly, although pulmonary inflammation induced by urban particulate matter can alter endogenous fibrinolysis (increased PAI-1) through a TNF-α-mediated process, it activates coagulation via a distinct IL-6-mediated pathway [7, 39, 40]. The mechanism responsible for DQ12-induced alteration of fibrinolytic function has not been identified, but may be related to the ability of particulate exposure to stimulate cytokine release from inflammatory cells [41] or release of an as yet unidentified mediator into the circulation [42].

Since our data suggest that alteration of endogenous fibrinolysis cannot solely account for acceleration of thrombosis, we addressed the role of platelet activation. Instillation of polystyrene particles increased platelet activation [43] and platelet-granulocyte aggregation in experimental animals [14]. The increase in platelet-monocyte aggregation 6 h after IT instillation of DEP in our investigation coincided with accelerated thrombus formation. This is consistent with previous work [3], and with a clinical study from our group [9]. In contrast, exposure to CB or DQ12 quartz had no effect, paralleling the pattern of thrombus effects across these particles. Thus, in contrast to inflammation and altered fibrinolysis, platelet activation was only seen following exposure to the particle (DEP) that accelerated thrombosis.

### Accelerated thrombosis without inflammation following i.v. injection of DEP

In addition to the role of inflammation, the cardiovascular consequences of particle exposure have also been attributed variously to alteration of the autonomic nervous system, translocation of particles from the lung into the systemic circulation, and oxidative stress [3]. Particle translocation has attracted attention since direct (i.v.) administration of particles to the blood increases thrombosis in vivo [13]. This may be due to direct effects on components of the cardiovascular system since ex vivo administration of DEP to blood accelerates clot formation [13]. We addressed the proposal that DEP could accelerate thrombus formation by direct interaction with the cardiovascular system using i.v administration of DEP (0.5 mg/kg) or control (CB) particles. The dose selected was based on previous i.v. exposures in rats (0.01-0.5 mg/kg; [21, 23, 31]. As expected i.v. injection of particles did not cause pulmonary inflammation.

Enhanced thrombus formation 2 h after i.v. injection of DEP is consistent with the proposal that this response is not secondary to inflammation. Interestingly, injection of CB (which also accelerated thrombus formation) did increase plasma CRP adding to the evidence that CB is not an “inert” control particle [41, 44]). This suggests that the nanocarbon core of injected DEP contributes to its pro-thrombotic action. This may be through direct interaction with the endothelium or generation of free radicals [45]. Certainly, CB (which models the nanocarbon core of DEP) alters endothelial cell function [41, 44], including inhibiting t-PA expression and protein production in cultured HUVECs [46]. The reduced t-PA:PAI-1 ratio observed after injection of DEP or CB suggests that alterations in endogenous fibrinolysis contribute to accelerated thrombosis following i.v. administration.

The use of cultured HUVECs demonstrated that DEP can alter endogenous fibrinolysis through direct interaction with the vascular endothelium. In the current investigation there was a pattern for lower thrombin-induced release of t-PA in cultured endothelial cells after exposure to DEP but this did not achieve significance [47]. However, our results are consistent with the suggestion that DEP can alter the endogenous fibrinolytic system by direct interaction with endothelial cells. Whether this occurs in vivo is debatable since the concentrations of DEP used were high and unlikely to be experienced by endothelial cells following pulmonary or systemic administration.

Particle administration to the blood in vitro directly activates platelets [5, 13, 43]. Since nanoparticles may translocate into the systemic circulation [48], it remains possible that inhaled particles could interact directly with platelets to accelerate thrombosis. In support of this notion, a reduction in platelet number (6 h) following i.v. injection of DEP in rats has been attributed to removal of aggregated platelets from the circulation [23]. Furthermore, the pro-thrombotic response to DEP has been linked to changes in platelet function [11, 13, 21, 49]. As with IT instillation, i.v. injection increased platelet-monocyte interaction, co-incident with accelerated thrombosis. Because both DEP and CB have been shown to be internalised by platelets [50], the ability of DEP to activate platelets is likely to be mediated by chemical species on the surface of DEP. Further studies are required to more precisely determine the mechanisms of DEP-induced platelet activation, including the role of platelet surface makers and glycoproteins [49, 50], interaction with circulating leucocytes and the blood vessel wall [51], changes in catecholamines [52] and the alterations in other blood constituents in susceptible models [53, 54]. Overall, these findings suggest that platelet activation, independent of pulmonary or systemic inflammation, contributes to the accelerated thrombus formation induced by DEP.

### Limitations

Identifying the mechanisms responsible for particle-induced acceleration of thrombus formation has proved challenging despite considerable research effort. The current study suggests a mechanism independent of systemic and pulmonary inflammation. It was designed to compare DEP suitable control particles, showing that particle induced inflammation does not necessarily increase thrombosis. As with all investigations, however, consideration must be given to potential limitations. An alternative approach to addressing this mechanism would be to use anti-inflammatory drugs or to study DEP-induced thrombosis in transgenic animals with impaired inflammatory pathways. The doses/exposures used in the experiments described here are higher than would be expected in the real world and are designed to provide conceptual insight into the mechanisms involved. They are also, however, single exposures in healthy animals; it is possible that chronic exposure of lower doses could be detrimental and may also have more effect in patients with underlying cardiovascular and/or respiratory disease. Furthermore, the magnitude of the effects produced is striking, suggesting that even lower levels could produce prominent effects in the blood. Blood thrombogenicity plays a key role in cardiovascular health and small imbalances towards increased clotting are associated with marked increases in the risk of cardiovascular events in patients with coronary artery disease and stroke.

## Conclusions

This study has used comparison with control particles and different routes of administration to demonstrate that DEP-mediated acceleration of thrombosis in vivo is independent of systemic and pulmonary inflammation. DEP directly altered components of the endogenous fibrinolytic system in endothelial cells but these changes alone could not account for the acceleration of thrombus formation. In contrast, platelet activation in response to DEP did not require pulmonary or systemic inflammation and may play an important role in increased thrombus formation. Although nanoparticle translocation is possible [48, 55–57], it seems unlikely that it would result in sufficient exposure to account for the acute acceleration of thrombus following instillation or inhalation [58]. Furthermore, similar changes in fibrinolytic function have been reported following pulmonary administration of DQ12 quartz, a particle considered too large to translocate [19, 20]. It is possible that this DEP-induced increase in thrombosis contributes to the increased cardiovascular disease risk in individuals exposed to vehicle emissions but whether this is the case remains to be determined.

## Methods

### Particle suspensions

Diesel exhaust particles (DEP; 1 mg/mL; Standard Reference Material 2975; National Institute of Standards and Technology (NIST) Gaithersburg, MD, USA), nano-Carbon Black (CB; 1 mg/mL; Printex90, Degussa, Germany) and quartz microparticles (0.25 mg/mL; DQ12, IUF, Germany) were suspended in sterile 0.9 % saline (in vivo experiments) or culture medium (EGM-2; Lonza Walkersville, USA; in vitro experiments) and dispersed by sonication (5 min; 70 % power, 5 cycles/s; Status Homogenisers, Philip Harris Scientific, UK). Suspensions were prepared immediately before use and were endotoxin free (Limulus Amebocyte Lysate assay, Cambrex, USA; [41]; data not shown). CB lacks the organic and metal constituents of DEP and represents a ‘clean’ carbon particle of a similar primary particle size to DEP. DQ12 quartz particles induce pulmonary inflammation but do not translocate from the lung due to their large particle diameter (~1 μm; [20]).

### Impact of particle administration on thrombosis in vivo

Animal work was performed under Home Office Licence (UK) in accordance with the Animals (Scientific Procedures) Act, 1986 and following local ethical committee review. Adult male Wistar Rats (Charles River, UK; 175–275 g) were housed under standard light cycle (lights on 08:00-20:00) and temperature (21–22 °C).

#### Administration of particle suspensions

Rats were anaesthetised by inhalation of isoflurane (Merial; Essex, UK) prior to administration of suspensions (0.5 mL) of DEP (1 mg/mL), CB (1 mg/mL) or DQ12 quartz (0.25 mg/mL; a dose previously shown to induce significant pulmonary inflammation) by intra-tracheal (IT) instillation [34]. A separate group of rats received 0.5 mL (0.5 mg/kg) DEP or CB suspensions, or vehicle (0.9 % saline), via tail vein intravenous (IV) injection.

#### Carotid artery thrombosis

Arterial thrombus was induced by application of ferric chloride (FeCl_3_; adapted from [59]). Briefly, carotid artery blood flow was measured in anaesthetised (isoflurane) rats using an ultrasonic flow probe (Transonic Systems Inc, Netherlands). FeCl_3_ solution (20 %) was applied topically to the carotid artery (10 min), arterial blood flow monitored, and the time to cessation of blood flow recorded.

#### Tissue retrieval

Rats were killed by exsanguination. Blood was collected from the abdominal aorta, citrated (10:1 in 3.8 % sodium citrate solution), and either analysed immediately (flow cytometry) or stored as plasma. Tissues (carotid artery, kidney, liver, lung) were processed for histological analysis. Serial sections (4 μm thick) were stained with hematoxylin and eosin.

### Bronchoalveolar lavage fluid collection and analysis

Bronchoalveolar lavage fluid (BALF) was obtained post-mortem by lavaging with sterile 0.9 % saline, centrifuged (180 *g*, 5 min, 4 °C) and the supernatant stored (−80 °C) for analysis. The cell pellet was re-suspended and a total cell count performed by an automatic cell counter (ChemoMetec A/S. Denmark). Additionally, cells (1 × 10^6^ cells per 300 μL) were re-suspended in saline containing 0.1 % bovine serum albumin, cytocentrifuged (300 rpm; 3 min) onto glass microscope slides (Superfrost Plus; VWR International Ltd, UK) and stained with Diff-Quick (Raymond Lamb, UK) for differential cell counting [19]. Lactate dehydrogenase (LDH) was measured using the Cytotoxicity Detection Kit (Roche Diagnostics, UK), according to manufacturer’s instructions.

### Pro-inflammatory cytokines in the lungs and systemic circulation

Plasma and BALF were assessed for inflammatory markers (interleukin (IL)-6, C reactive protein (CRP) and tumour necrosis factor alpha (TNFα)) by ELISA (R&D Systems, UK) to manufacturer’s instructions, using an automated Triturus ELISA analyser (Grifols, Spain).

### Endogenous fibrinolysis

Plasma samples were assessed for t-PA and active PAI-1 using commercially-available ELISA kits (Patricell Ltd, UK).

### Platelet-monocyte aggregation

Platelet-monocyte aggregates were measured by flow cytometry using fluorescein isothiocyanate (FITC)-labelled anti-rat CD42d antibody [60] and phycoerythrin (PE)-labelled hamster anti-rat CD61 [61]. PE-labelled hamster IgG1κ was used as a negative isotype control (antibodies from BD Biosciences, UK). Samples were analysed by BD FACscan flow cytometer equipped with CellQuest software for data acquisition and FlowJo software for data analysis. Platelet-monocyte aggregates were defined as double positive for CD42d and CD61.

### The endogenous fibrinolytic pathway in HUVECs

Human umbilical vein endothelial cells (HUVECs; Promocell, Germany; Passage ≤6) were maintained in EGM-2. Confluent HUVECs were exposed to DEP (10–150 μg/mL) or vehicle (medium alone) for 2–24 h. Following exposure, cells and supernatants were stored (−80 °C) for subsequent analysis. Alternatively, DEP suspensions were removed and the cells stimulated with thrombin (0.1–1 U/mL; 24 h) before harvesting. The effect of DEP on cell viability was assessed by LDH assay (Cyotoxicity Detection kit; Roche Diagnostics, UK).

Concentrations of t-PA (antigen and activity) and PAI-1 (activity) in HUVEC supernatants were measured by ELISA, to manufacturer’s instructions (t-PA Combi Actibind ELISA kit and TECHNOZYM® PAI-1 Actibind® ELISA kit; Technoclone Ltd, UK). RNA was extracted from HUVECs and reverse transcribed to cDNA in order to measure t-PA and PAI-1 expression by real time polymerase chain reaction (PCR). Real time PCR primers (Invitrogen; UK; Additional file 3: Table S1) and probes (Roche Universal Probe library) were designed by the Roche UPL Design Centre (Roche, UK) using the species-specific gene and nucleotide sequences. Samples were analysed using a Roche LightCycler 480 and normalised to the housekeeping genes GAPDH and β-actin. Additional reagents were purchased from Sigma-Aldrich (UK).

### Statistics

Data are expressed as mean ± s.e.mean, *n* = 6 unless otherwise indicated. Results were analysed using GraphPad Prism software. Analyses were performed using Student’s unpaired, two-tailed *t*-test, one-way ANOVA followed by Tukey’s multiple comparison post-test or two-way ANOVA with a Bonferroni post-test. Equality of variance was confirmed using an F test or Kolmogorov-Smirnov test. Significance was assumed when *P* < 0.05.

## Additional files

Additional file 1: Figure S1. Intravenous administration of DEP does not increase systemic CRP or pulmonary pro-inflammatory cytokines. Intravenous injection of DEP (black columns) or CB (light grey columns) did not increase bronchoalveolar lavage fluid concentrations of (i) TNF-α or (ii) C-reactive protein (CRP) when compared to saline (white column) collected 2 h after injection. Levels of IL-6 were below the limit of detection. (iii) Systemic inflammation was assessed by measuring CRP in plasma taken from rats 2 h (the time-point at which thrombus formation was increased by DEP) after intravenous injection of diesel exhaust particulate (DEP) or carbon black (CB). CB (light grey column), increased plasma concentrations of CRP whereas DEP did not (black column). Data are mean ± s.e.mean (*n* = 6) and were compared using Student’s unpaired *t*-test ((i) & (ii)) or one-way ANOVA (iii); ns = not significant ***P < 0.001 compared with saline-treated control. (TIF 55 kb)

Additional file 2: Figure S2. Assessment of cytotoxicity in endothelial cells exposed to DEP suspensions. Cell death was induced in human umbilical vein endothelial cells by exposure (24 h) to (a) H_2_O_2_ (1–1000 μM; *n* = 1) or (b) Tween 20 (0.1–10 %; *n* = 1) (c) but not by diesel exhaust particles (DEP; 10–80 μg/ml) after 2 (red), 6 (blue) 16 (green) or 24 (purple) hours incubation. Data are mean ± s.e.mean (*n* = 6). (TIF 81 kb)

Additional file 3: Table S1. Real time polymerase chain reaction primers. (TIF 53 kb)

## Abbreviations

°C: Degrees celsius
ANOVA: Analysis of variance
CB: Carbon black
cDNA: Complementary deoxyribonucleic acid
CRP: C reactive protein
DEP: Diesel exhaust particles
DQ12: Quartz particles
ELISA: Enzyme-linked immuno sorbent assay
FACS: Fluorescence-activated cell sorting
FeCl_3_: Ferric chloride
FITC: Fluorescein isothiocyanate
g: gram
GAPDH: Glyceraldehyde 3-phosphate dehydrogenase
h: Hours
HUVECs: Human umbilical vein endothelial cells
i.v.: Intra-venous
IL-6: Interleukin-6
IT: Intra-trachael
kg: Kilogram
LDH: Lactate dehydrogenase
LPS: Lipopolysaccharide
mg: Milligram
min: Minute
mL: Millilitre
mm: Millimeter
NIST: National Institute of Standards and Technology
PAI-1: Plasminogen activator inhibitor-1
PBS: Phosphate buffered saline
PCR: Polymerase chain reaction
PE: Phycoerythrin
PM: Particulate matter
RNA: Ribonucleic acid
rpm: Revolutions per minute
SEM: Standard error of the mean
TNFα: Tumour necrosis factor-α
t-PA: Tissue plasminogen activator
U: Unit
Veh: Vehicle
μg: Microgram
μm: Micrometer
