# Supplementary figures and images for: Platelet activation independent of pulmonary inflammation contributes to diesel exhaust particulate-induced promotion of arterial thrombosis
Source: Part Fibre Toxicol. 2016 Feb 9;13:6. doi: 10.1186/s12989-016-0116-x (PMC4746929; doi:10.1186/s12989-016-0116-x)

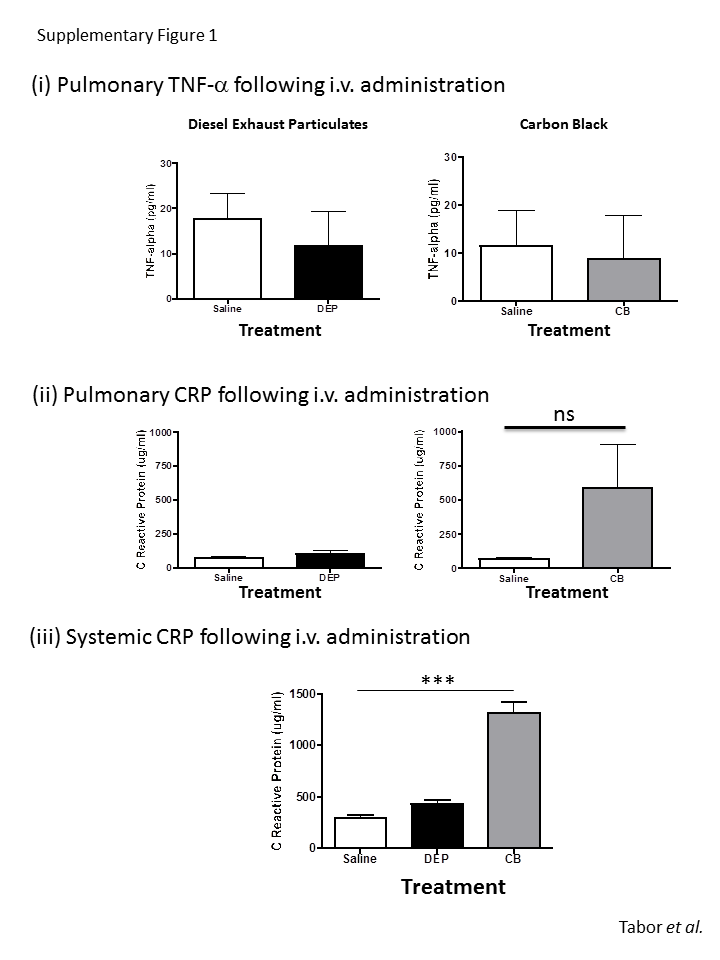

Supplement: Supplementary file 1 — Intravenous administration of DEP does not increase systemic CRP or pulmonary pro-inflammatory cytokines. Intravenous injection of DEP (black columns) or CB (light grey columns) did not increase bronchoalveolar lavage fluid concentrations of (i) TNF-α or (ii) C-reactive protein (CRP) when compared to saline (white column) collected 2 h after injection. Levels of IL-6 were below the limit of detection. (iii) Systemic inflammation was assessed by measuring CRP in plasma taken from rats 2 h (the time-point at which thrombus formation was increased by DEP) after intravenous injection of diesel exhaust particulate (DEP) or carbon black (CB). CB (light grey column), increased plasma concentrations of CRP whereas DEP did not (black column). Data are mean ± s.e.mean (n = 6) and were compared using Student’s unpaired t-test ((i) & (ii)) or one-way ANOVA (iii); ns = not significant ***P < 0.001 compared with saline-treated control. (TIF 55 kb) [file 12989_2016_116_MOESM1_ESM.tif]

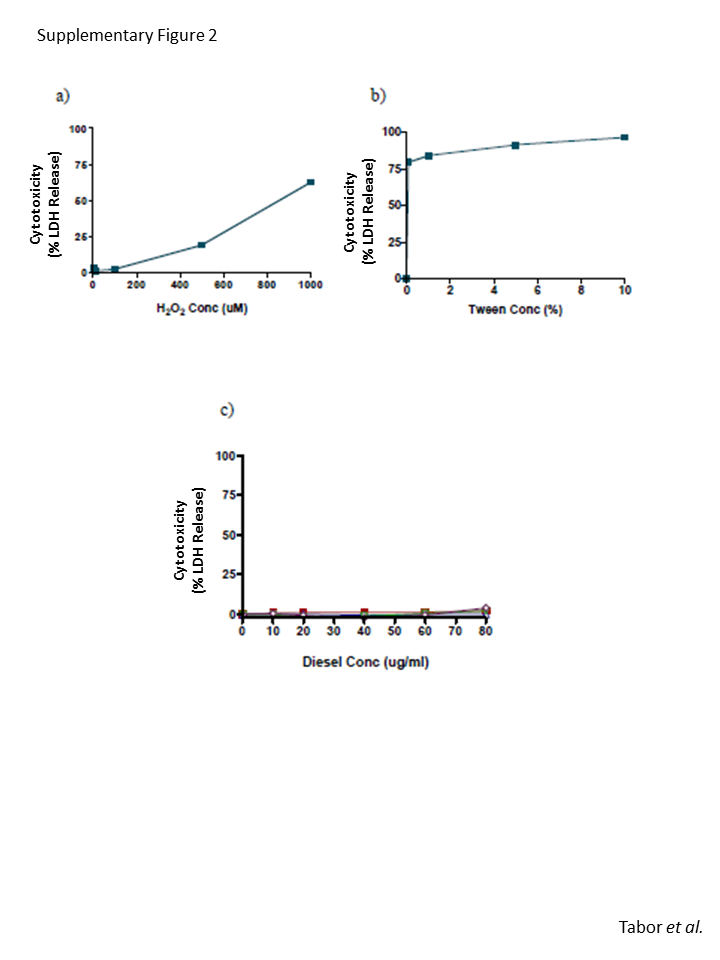

Supplement: Supplementary file 2 — Assessment of cytotoxicity in endothelial cells exposed to DEP suspensions. Cell death was induced in human umbilical vein endothelial cells by exposure (24 h) to (a) H2O2 (1–1000 μM; n = 1) or (b) Tween 20 (0.1–10 %; n = 1) (c) but not by diesel exhaust particles (DEP; 10–80 μg/ml) after 2 (red), 6 (blue) 16 (green) or 24 (purple) hours incubation. Data are mean ± s.e.mean (n = 6). (TIF 81 kb) [file 12989_2016_116_MOESM2_ESM.tif]

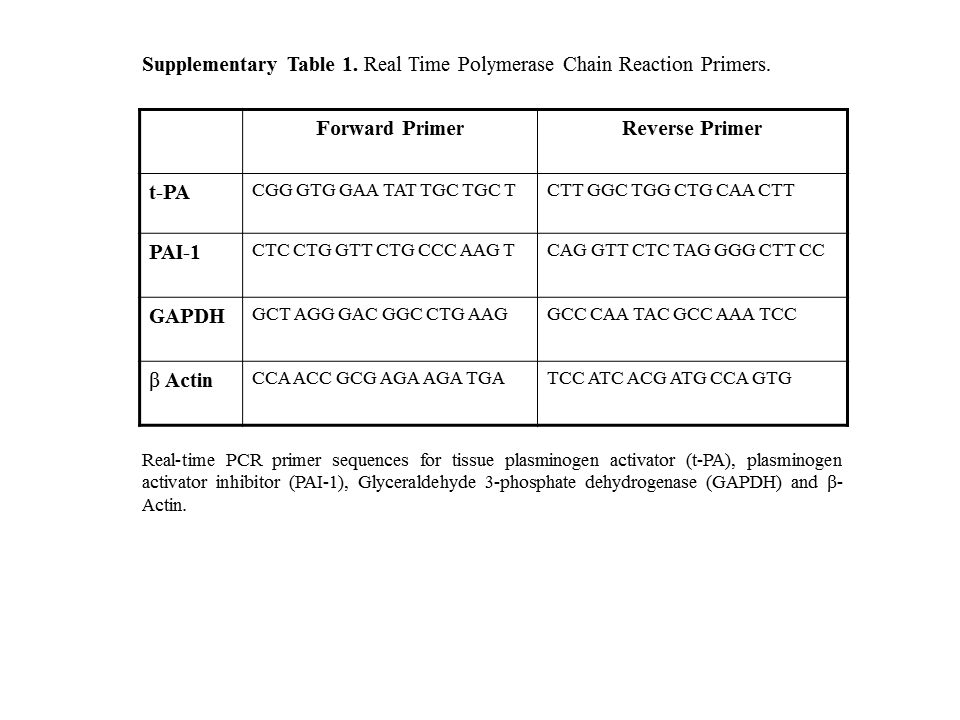

Supplement: Supplementary file 3 — Real time polymerase chain reaction primers. (TIF 53 kb) [file 12989_2016_116_MOESM3_ESM.tif]
